# An Insight into a Sustainable Removal of Bisphenol A from Aqueous Solution by Novel Palm Kernel Shell Magnetically Induced Biochar: Synthesis, Characterization, Kinetic, and Thermodynamic Studies

**DOI:** 10.3390/polym13213781

**Published:** 2021-10-31

**Authors:** Kamil Kayode Katibi, Khairul Faezah Yunos, Hasfalina Che Man, Ahmad Zaharin Aris, Mohd Zuhair Mohd Nor, Rabaah Syahidah Azis

**Affiliations:** 1 Department of Agricultural and Biological Engineering, Faculty of Engineering and Technology, Kwara State University, Malete 23431, Nigeria; kamil.katibi@kwasu.edu.ng; 2 Department of Biological and Agricultural Engineering, Faculty of Engineering, University Putra Malaysia, Serdang 43400, Selangor, Malaysia; hasfalina@upm.edu.my; 3 Department of Food and Process Engineering, Faculty of Engineering, University Putra Malaysia, Serdang 43400, Selangor, Malaysia; zuhair@upm.edu.my; 4 Department of Environment, Faculty of Forestry and Environment, University Putra Malaysia, Serdang 43400, Selangor, Malaysia; zaharin@upm.edu.my; 5 Material Processing and Technology Laboratory (MPTL), Institute of Advance Technology (ITMA), University Putra Malaysia, Serdang 43400, Selangor, Malaysia; 6 Department of Physics, Faculty of Science, University Putra Malaysia, Serdang 43400, Selangor, Malaysia; rabaah@upm.edu.my; 7 Materials Synthesis and Characterization Laboratory (MSCL), Institute of Advanced Technology (ITMA), University Putra Malaysia, Serdang 43400, Selangor, Malaysia

**Keywords:** neat biochar, palm kernel shell, magnetic biochar, adsorption mechanism

## Abstract

Recently Bisphenol A (BPA) is one of the persistent trace hazardous estrogenic contaminants in the environment, that can trigger a severe threat to humans and environment even at minuscule concentrations. Thus, this work focused on the synthesis of neat and magnetic biochar (BC) as a sustainable and inexpensive adsorbent to remove BPA from aqueous environment. Novel magnetic biochar was efficiently synthesized by utilizing palm kernel shell, using ferric chloride and ferrous chloride as magnetic medium via chemical co-precipitation technique. In this experimental study, the influence of operating factors comprising contact time (20–240 min), pH (3.0–12.0), adsorbent dose (0.2–0.8 g), and starting concentrations of BPA (8.0–150 ppm) were studied in removing BPA during batch adsorption system using neat biochar and magnetic biochar. It was observed that the magnetically loaded BC demonstrates superior maximum removal efficiency of BPA with 94.2%, over the neat biochar. The functional groups (FTIR), Zeta potential, vibrating sample magnetometer (VSM), surface and textural properties (BET), surface morphology, and mineral constituents (FESEM/EDX), and chemical composition (XRD) of the adsorbents were examined. The experimental results demonstrated that the sorption isotherm and kinetics were suitably described by pseudo-second-order model and Freundlich model, respectively. By studying the adsorption mechanism, it was concluded that π-π electron acceptor–donor interaction (EAD), hydrophobic interaction, and hydrogen bond were the principal drives for the adsorption of BPA onto the neat BC and magnetic BC.

## 1. Introduction

Recently, the safety and quality of drinking water has been problematic owing to rapid urban development induced by human activities. The upsurge of some micro-contaminants identified as endocrine disrupting compounds (EDCs) has elicited increasing concerns over the supply of safe and clean drinking water [1,2,3]. Particularly, Bisphenol A (BPA; 2,2-bis(4-hydroxyphenyl)propane) is an intermediate and highly essential raw material highly utilized in the manufacture of several polycarbonate plastics and epoxy resins (plastic component), food cans, polyester fibers, and thermal paper as well as other materials in industry, thus it frequently emerges in numerous products for daily use, such as electronic equipment, water-pipes, toys, or paper [4,5]. Globally, BPA is classified among the most ever-present and most extensively produced synthetic chemical compounds in manufacturing today, with more than three million tons generated annually [6]. Markedly, the manufacture and sale of plastics comprising BPA has been prohibited by the Brazilian government since 2012, owing to its environmental universality, elevated industrial output, and toxicological consequence, and it has been classified as an important contaminant in water purification in many regions and nations [7]. Furthermore, exposure to BPA is through tableware and polycarbonate bottles, including those employed for new-born formula milk, and by means of epoxy resin coatings inside beverage and food containers, dental fissure sealants, adhesives, epoxy-based surface coatings, canned goods, household dust, and printing inks [5,6] and massively released into the water environment [8]. BPA in plastic containers can hydrolyse and pollute groundwater via landfill leachate [9]. Risen plastic industries trigger profuse discharge of BPA into the environment and it is detected in drinking and surface water [10]. More unfortunately, BPA has undesirable ecological impacts that are associated with dysfunction of the hormone system in animals and humans, even at very minuscule concentration. Specifically, an estrogenic action of BPA can provoke cancer [11,12,13], neurological challenges, diabetes, tumors, obesity, immune effects, heart disease in humans, damaged reproductive function, disturbance of the normal hormone functions, and undesired physiological state in animals and humans, biomagnification, and bioaccumulation through food chain or food web in human beings [14,15]. Besides, the dysfunction and damages caused by BPA are long-term [16]. Therefore, releasing water-containing BPA into waterways without adequate treatment poses a damaging impact on the environment (humans and aquatic) [17]. Owing to the frequent extensive utilization of BPA-based products by humans with their associated negative impacts on public health and ecosystem, hence, the removal of BPA became unavoidable. Extensive efforts have been employed towards extenuating the undesirable effects and environmental hazards by reducing the contaminants’ concentration using various treatment technologies, including precipitation, coagulation, activated sludge, biological filter, and constructed wetlands, with inadequate effect on BPA removal [18,19,20,21]. Some relatively advanced treatment techniques, such as ozonation and advanced oxidation [22,23], Fenton oxidation [24], membrane technology [25,26], photocatalysis degradation [27], enzymatic degradation [28,29], and adsorption technique utilizing activated carbon [30,31,32] exhibit a good BPA removal from wastewater and drinking water. Yet, the above treatment approaches still have significant drawbacks, such as high capital and maintenance cost, complex treatment procedures with increased operating expenses, costly capital expenses (CAPEX), generation of toxic by-products which hamper their application in developing nations [33]. Noticeably, among the aforementioned water-treatment approaches, adsorption technique is found more suitable, environmentally friendly, relatively economical, robust, and simple, and it could efficiently be employed in large-scale applications devoid of generating by-products in the environment [34,35]. Adsorption method has attracted wide attention and has been extensively considered as a promising technology for removing BPA pollutant during the past two decades, due to its ease of configuration and application, high efficiency, insensitivity to poisonous compounds, low cost, and comparatively small footprint as compared to other water-treatment methods [31].

Several adsorbents are available, however, carbon materials have been reported to perform remarkably owing to their several benefits, such as enormous surface area, superior stability, and outstanding removal efficiency [36]. Activated carbons generated from biomass wastes are often used as adsorbent during the adsorption process for the removal of emerging pollutants [37,38], since it can efficiently and rapidly remove BPA as a result of its abundant surface area as well as adsorptive capacity; but, its preparation is highly expensive. Thus, various studies have considered reliable resources, low-cost, and environment-friendly materials as alternative adsorbents [38,39]. Thus far, traditional carbonaceous materials, such as BC, activated carbon, graphene oxide together with its derivates, and carbon nanotubes, have been studied as promising adsorbent materials for hydrophobic organic pollutants, including pharmaceutical compounds and EDCs [40]. Furthermore, biochar as an adsorbent has porous structure comparable with activated carbon, which is the most generally utilized and effective sorbent for the removal of various contaminants from water globally [41].

Biochar, a major pyrogenic by-product obtained from the complete or partial pyrolysis of naturally plenteous biomass under oxygen-deficient conditions [42], has several macropores, hence it could attain a superior adsorption capacity [43]. More importantly, BC exhibits superior unique properties, such as augmented surface functional groups, porous structure, abundant specific surface area, eco-friendly, low-cost, ample inherent mineral components, high cation exchange capacity, and, notably, is efficient in the removal of various hydrophobic and hydrophilic organic pollutants owing to its high aromaticity and hydrophobicity [40,44,45]. These properties have made BC to be the best and most promising precursor over other adsorbent materials for various contaminants removal. Synchronically, after pyrolysis, BC can be reapplied to sorb contaminants in water; thus, it has abundant utilization value [46]. However, the minuscule particle size of BC in addition to its lower density makes its regeneration, separation, and recovery more problematic after adsorption, and this could undermine its recycling capacity and industrial applications. In order to subdue these difficulties, few studies have considered synthesis of magnetically recyclable biochar (magnetic biochar) via the implantation of iron oxide (Fe_3_O_4_ and Fe_2_O_3_) [47]. For instance, Lu et al. [48] studied the removal of BPA using N-doped ulva prolifera (marine macroalgae). Over 90% of BPA was eliminated and the sorption capacity of 9.19 mg/g was attained within 4 hrs. In another application, Heo et al. [49] synthesized CuZnFe_2_O_4_ composite biochar using bamboo to enhance the removal of BPA and sulfamethoxazole (SMX) from aqueous solution. The adsorption capacity of 263.2 mg/g was recorded when CZF–biochar adsorbent was applied to remove BPA. It was observed that the sorption capacity was improved for SMX and BPA when CZF nanomaterials were impregnated on the surface of biochar. Furthermore, Wang et al. [16] use grapefruit peel biochar to remove BPA under variable adsorbent dosage, pH, and contact time. The authors reported that pH has a significant influence on the adsorption of BPA and that almost 100% BPA removal was achieved at a pH (6).

In Malaysia, the agro-industrial sector generates a substantial volume of biomass solid wastes, predominantly from palm oil mills which produce the huge expanse of biomass, including palm kernel shells (PKS), empty fruit bunches, oil palm fibers, and palm oil mill effluents [50]. Malaysia recorded the largest export of 19.9 million tonnes (mt) from oil palm biomass residues in 2017 [51]. With the expansion of palm oil production in Malaysia, the volume of residue generated has correspondingly risen. Approximately 50–70 tonnes of biomass residues could be produced from a hectare of oil palm plantation [52]. Thus, the palm oil industry is presently generating around 50 mt of dry oil palm residues annually and attain 100 mt per year by 2020 [53]. Notably, PKS has the maximum commercial consumption value as compared to other oil palm biomass [54]. Besides, the specific characteristics of PKS-BC (palm kernel shell biochar), particularly its porous structure, enhancement of functional groups, its huge specific surface area, and improved mineral component, make it suitable for use as adsorbent material [42]. Despite this huge potential, PKS is considered as biomass waste, generating superfluous waste of resources and environmental nuisance [55].

There is still a lack of reports on the adsorption of BPA using PKS magnetic BC, as well as its adsorption mechanism which is yet to be explored and needs to be investigated. In view of this highlight, PKS biomass waste that is available in huge quantities in Malaysia was applied as a precursor for the synthesis of magnetic BC as a sustainable and inexpensive adsorbent for the removal of BPA from aqueous solutions via adsorption procedure.

Magnetic BC was synthesized using the chemical co-precipitation method. A batch adsorption test was employed to evaluate the maximum adsorption capacity of BC produced from PKS. The effects of essential factors, in particular contact time, adsorbent dosage, ionic strength, initial BPA concentration, and pH, on the adsorption of BPA by NBC (neat biochar) and MBC (magnetic biochar) were studied. The adsorption mechanism of BPA by synthesized BC was examined together with the analysis of the NBC and MBC, to boost the study of the adsorption of BPA by NBC and MBC. The adsorption isotherm was stimulated via Freundlich and Langmuir models. The adsorption kinetics and reusability of the novel synthesized PKS-BC were also investigated.

## 2. Materials and Methods

### 2.1. Materials and Chemicals

All chemicals: Bisphenol A (99% purity) reagent, ferric chloride hexahydrate (FeCl_3_6H_2_O), ferrous sulfate heptahydrate (FeSO_4_7H_2_O), and sodium hydroxide (NaOH) (AR) utilized in this study were of analytical grade and purchased from Sigma-Aldrich (West Chester, PA, USA) and applied without additional purification. Distilled water (DW) was utilized for the preparation of all the aqueous solutions during the entire experiment. The BPA solution utilized in the experiment was carefully prepared prior to each experiment to avoid possible microbial degradation. The chemical and physical properties of BPA are presented in Table 1. The PKS biochar was obtained from AMR Environmental Sdn Bhd, located at Johor, Bahru, Malaysia.

### 2.2. Synthesis of Magnetic Biochar

The impregnation of biochar with magnetite using co-precipitation of ferric and ferrous salts on carbonaceous materials method was according to the procedure employed in previous studies [16]. Initially, the PKS-BC sample was grounded and modified using high energy ball milling (HEBM) for 3 hrs to achieve a nano-sized biochar. The obtained ball-milled powdered BC was passed through 50–63 µm sieves and washed with distilled water and ethanol three times. Afterwards, the PKS-BC was oven-dried overnight at 110 °C. The obtained dried BC was collected in a bottle container, sealed, and labelled as NBC and kept in a desiccator for further use. In the next phase, 8.5 g of the dried PKS-BC was soaked in a 100 mL of FeCl_3_ of 0.25 mol·L^−1^ and 0.125 mol·L^−1^ of FeSO_4_ solution and uniformly mixed via the magnetic stirrer. The mixtures were subjected to heat at 60 °C with constant stirring to ensure that Fe^3+^ and Fe^2+^ could saturate into BC and later allowed to cool to 40 °C. Subsequently, 100 mL of 1 mol·L^−1^ of sodium hydroxide (NaOH) solution was dropwisely added to the solution and stirred until the pH attained the range of (10–11), and the colour of the solution transform from brownish green to black. Fe_3_O_4_ precipitation was formed on the surface of carbon materials under this alkaline condition. Upon the completion of the reaction, the NaOH-impregnated biochar solution was adequately stirred for 60 min to achieve better dispersion and homogenous mixture. The resultant mixture was separated by an external magnet and rinsed with distilled water until the solution pH closed to 7 and then oven-dried at 70 °C for 12 h. The final modified BC sample was grounded and collected into a bottle container, sealed, and labelled as MBC and kept in a desiccator thereafter.

### 2.3. Characterization of Neat and Magnetic Biochar

The magnetic properties and magnetization curve of MBC was determined using a vibrating sample magnetometer (Lakeshore 7404, Westerville, OH, USA) with an applied field between −10,000 and 10,000 Oe at room temperature, from American Quantum.

Surface functional groups of the BC samples (before and after modification) were confirmed using Fourier transform infrared (FTIR) spectroscopy (Perkin Elmer, 1650 Spectrometer) within the scanned range of 400 to 4000 cm^−1^ using the attenuated total reflection method.

The textural properties such as surface area, pore volume, and pore size distribution of the samples were determined by the standard N_2_ physisorption procedures using Micrometrics analyzer (Tristar II Plus model) and determined in line with the Brunauere Emmentte Teller (BET) technique with degassed temperature of 350 °C.

X-ray diffraction (XRD) was used to analyse the phase and chemical composition of the prepared BC adsorbents at Cu Kα radiation (2θ spectrum = 20–80°; phase = 0.05° 2θ; time per step = 0.2 s) via X-ray powder diffractometer (Rigaku MiniFlex 600). The compositions and surface morphologies of the samples were detected by field emission scanning electron microscopy (FESEM, Zeiss ULTRA 55) and energy dispersive spectroscopy (EDS, Bruker/Quanta 200), Westerville, OH, USA).

Also, the pH of BC was assessed viz: BC was blended with distilled water at (1:10) mass ratio, agitated magnetically for 30 min, and subsequently kept for 60 min. Afterwards, the pH of BC was determined by a pH5S Spear pH tester (T531009086, Shanghai, China).

### 2.4. Analysis of Surface Chemistry of Biochar (pH_pzc_)

The surface chemistry of neat and magnetically-modified biochar was performed experimentally using the pH at the point of zero charge according to the procedure of [56] with slight modifications. Ten samples of varying pH (2.0–11.0) were prepared using a 0.01 M aqueous solution of sodium acetate (C_2_H_3_NaO_2_) as the base electrolyte. The pH of the solution was adjusted using either 0.1 M of NaOH or 0.1 M HCl aqueous solution. Then, 0.1 g of magnetic and neat samples of BC were carefully added to each 20 mL of the prepared solutions in a conical flask placed in a swing agitator at a speed of 120 rpm at 298 K room temperature and stirred for 48 h. Afterwards, the resultant supernatant was then decanted, and its pH was determined. The value of pH_pzc_ was computed from a plot of pH of the initial solution against pH of the supernatant [57,58]. Thus, the zeta potentials of neat biochar (NBC) and magnetic biochar (MBC) were examined via zeta potential analyzer (Zetasizer Nano Plus 3, Zeta/nano particle analyzer, Malvin, Austin, TX, USA).

### 2.5. Adsorption Experiment

Definite amount of NBC and MBC adsorbents (50.0 mg) and 125.0 mL of BPA solution were weighed and added to a set of 250 mL; Erlenmeyer conical flasks sealed externally using aluminium foil to avoid probable photodegradation. The mixture was then placed on an orbital shaker (HY-8, Shanghai, China) and agitated mechanically at 160 rpm at room temperature for 24 h to achieve equilibrium condition. Upon the completion of the agitation process and at pre-specified times, the solution was rapidly removed and filtered using 0.4-µm membrane and the absorbance of the supernatants were analyzed spectrophotometrically at a wavelength of 276 nm using a double beam UV-visible spectrophotometer (Shimadzu UV-1800, Kyoto, Japan) with a pair of 10 mm matched quartz cells, and the concentrations were converted into the established BPA calibration (standard) curve [59,60].

In the single-variable experiment, the effects of adsorbents (BC) doses (0.2–0.8 g; BPA = 20 ppm), pH range were adjusted by 0.01 mol/L HCl and 0.01 mol/L NaOH solution (3.0–12.0; BPA = 20 ppm), contact time (20–240 min; BPA = 20 ppm), ionic strength (Na^+^ concentration: 0, 0.2, 0.4, 0.6, 0.8, and 1.0 mol/L, BPA = 20 ppm); initial BPA concentrations (8–150 ppm), and reaction temperature (298.15, 318.15, and 338.15 K), on the adsorption of BPA by NBC and MBC were studied. The amount of BPA adsorbed on BC adsorbent was determined using the following Equation (1):

(1) $$q_{t}=\frac{\left(C_{S}-C_{t}\right)}{M}\times V$$

where, *C_s_* and *C_t_* are the starting and residual concentrations of BPA in mg/L, *M* is the mass of the adsorbent (g); and *V* represent the solution volume (L).

For the adsorption thermodynamic tests, 0.5 g of the adsorbent and 100 mL of BPA solution were added to 250 mL conical flask to study the adsorption thermodynamic of MBC for BPA. The mixtures were stirred in a temperature-regulated incubator shaker (Excella E24 Incubator shaker series, USA) at a speed of 150 rpm, pH (6) for 60 min adsorption time, and the adsorption temperature were varied between (298.15 K–338.15 K).

### 2.6. Statistical Analysis

For the adsorption experiment, each experimental samples and group were carried out in triplicate, and the average value of the data was considered. The removal efficiency of BPA (*Re%*) and quantity of BPA adsorbed (*q_e_*) onto BC adsorbent were determined.

The adsorption isotherms, kinetics (20–240 min), and thermodynamics (298.15, 318.15, and 338.15 K) were studied. Each test was carried out in triplicate, and the average results were considered.

### 2.7. Reusability and Regeneration of MBC

The reusability studies on the prospect of desorbing of BPA compounds from MBC are indispensable based on environmental and economical perspective, in addition to industrial applicability [61]. With the purpose of achieving regeneration of MBC, an efficient and green eluent was considered. The reusability of MBC at the starting BPA concentration of 20 ppm was assessed by rinsing BPA-ladened MBC with 0.1-M NaOH and distilled water and subjected to ultrasonication, and then followed by oven-drying at 60 °C overnight after magnetic field separation [49]. The MBC was repetitively used five times, and the values of *q_e_* was noted accordingly.

### 2.8. Adsorption Isotherm

Adsorption isotherm was employed to compute the quantity of adsorbed BPA on BC based on BPA concentration at a steady temperature. The application is based on standardizing the amount of BPA adsorbed by the mass of BC adsorbent and this confirms comparison with Isotherm models.

### 2.9. Langmuir Adsorption Isotherm

The Langmuir model relies on the assumption that uptake of BPA takes place on a homogenous surface via monolayer adsorption with no interaction between adsorbed materials. It also presumes that all sorption sites are ‘correspondingly active’, and the surface is robustly homogeneous [62]. Principally, the larger values of *R*^2^ signify the significance of the adsorption model for contaminants removal in water. The value of *R_L_* between 0 and 1 implies that the isotherm is advantageous. The isotherm is unsuitable if *R_L_* > 1, linear if *R_L_* = 1, irreversible if *R_L_* = 0, and favorable if *R_L_* lies between 0 to 1. Arithmetically, the Langmuir isotherm model is denoted in Equation (2):

(2) $$\frac{1}{q_{e}}=\frac{1}{K_{L}q_{max.}}\cdot \frac{1}{C_{e}}+\frac{1}{q_{max.}}$$

where *C_e_* represent BPA concentration at equilibrium (mg/L); *q_max._*(mg/g) is the maximum single-layer adsorption capacity of the BC adsorbent, *q_e_* (mg/g) is the quantity of BPA adsorbed; *K_L_* (mg/g) represent the Langmuir constant closely related to adsorption capacity for overall monolayer coverage; the intercept (1/*K_L_*), slope (1/*q_max._*), and *q_max_.* could be evaluated from a plot of 1/*q_e_* versus 1/*C_e_* for BPA adsorption onto NBC and MBC.

### 2.10. Freundlich Adsorption Isotherm

Freundlich isotherm model was employed to study the uneven distribution on the surface of the absorbent, which is heterogeneousness in the adsorption process [63]. The model indicates that the adsorption energy proportionally declines on the endpoint of the adsorption centres of an adsorbent [64]. The degree of correlation between adsorption and solution concentration relies on the adsorption intensity, n. The adsorption conditions can be chemical (n < 1), favourable physical process (n > 1) and favourable linear (n = 1). The *K_F_* and n are Freundlich constants and were found from the graph as the intersection and slope, respectively. Scientifically, the Freundlich isotherm model can be described in Equation (3). The graph of ln *q_e_* against ln *C_e_* provide a linear plot with an intercept log*K_F_* and slope 1/n, from which *R*^2^, *K_F_* and *n*, can be computed, respectively [65].

(3) $$\log q_{e}=\log K_{f}+\frac{1}{n}\log C_{e}$$

## 3. Results

### 3.1. Results and Discussion

#### Characterization and Analysis of Synthesized Biochar Adsorbents

The surface chemistry and morphological characterization of BC sorbents are excellent indicators of adsorption propensity. Hence, VSM, FTIR, FESEM, EDX, and BET surface analyses, together with the point of zero charge of the adsorbents (NBC and MBC) were also evaluated.

### 3.2. Morphological Analysis of Synthesized Biochar

**Figure 1:**
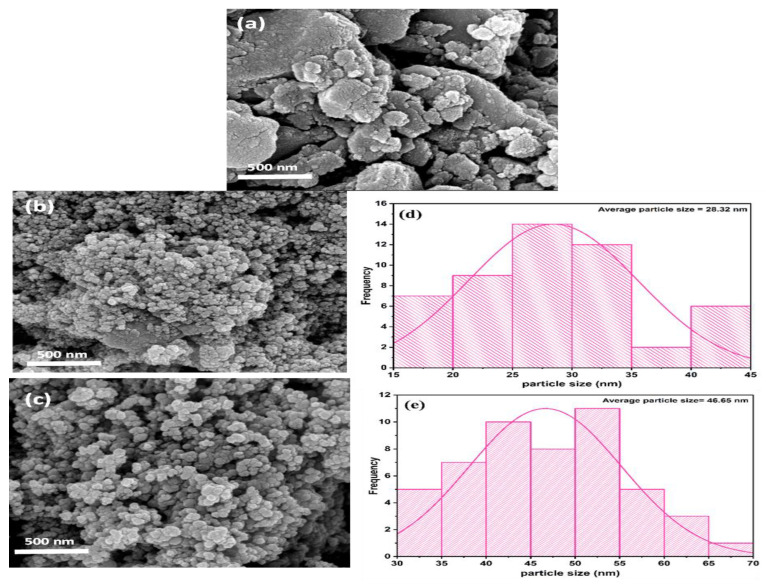
FESEM (field emission scanning electron microscopy) images of NBC (**a**), MBC before adsorption (**b**), magnetic biochar after adsorption (**c**), average particle size distribution before (**d**), and after adsorption (**e**).

FESEM analysis was conducted to examine the surface morphology of the BC samples. The presence of abundant pores were observed on the surface of the BC, which was substantiated by the FESEM micrographs (Figure 1a–c). Images of NBC, MBC (before and after adsorption) are described in Figure 1b,c. The FESEM analysis of the MBC (prior to adsorption and after adsorption) was performed to observe the morphological structure along with the particle size and size distribution of MBC, as presented in (Figure 1b–e), respectively. From Figure 1a–c, the images showed the particles obtainable are spherical in shape with a single uniform aggregate and small non-uniform agglomerates. In Figure 1a, larger particles with a porous and rougher surface were noticed for NBC. Also, the particles possess more cleavages with few connected to bulks. Furthermore, it can be observed from the FESEM micrographs (Figure 1a,b) of NBC and MBC, that the surface of NBC is relatively coarse and rough since the spherical shaped particles with particle size more than 28.32 nm were agglomerated and attached to each other and the pore form is not fully developed. Contrastingly, the surface of MBC is comparatively smooth, with substantial porous structure than NBC, and the surface is filled with numerous nano-iron oxide particles. The dispersal of Fe_3_O_4_ nanoparticles on the surface of BC is relatively uniform. The surface of MBC developed shinier and smoother surface after adsorbing BPA (Figure 1c). The average particle size of MBC prior to adsorption as depicted in Figure 1d was computed to be 28.32 nm. Conversely, after adsorption of BPA, the average particle size rose to 46.65 nm as showed in Figure 1e, confirming that the adsorption process was taking place.

### 3.3. Elemental Analysis of Synthesized PKS Biochar

**Figure 2:**
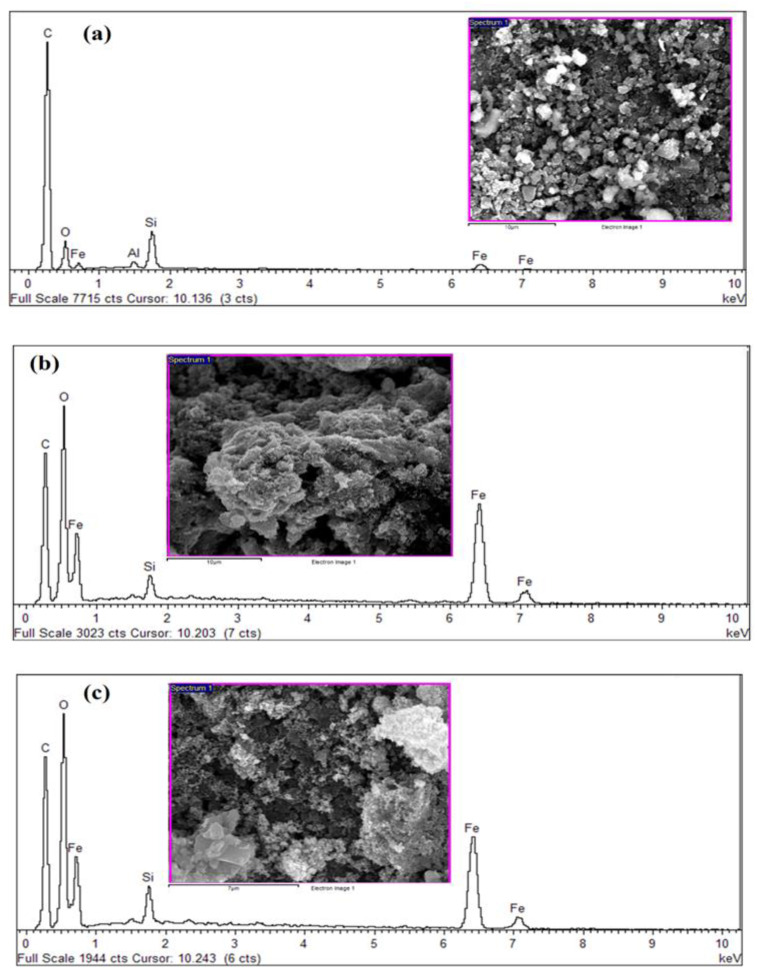
EDX spectra of the as-synthesized NBC (**a**), and MBC prior to adsorption (**b**), and after adsorption (**c**).

For this procedure, 1.0 g of each of the samples (NBC and MBC) was utilized for the EDX analysis. This test studied different component elements existing in each of the BC samples. The EDX test performed reveal the elemental compositions and distribution both in the NBC and MBC. Figure 2 presents the elemental analysis of the NBC and MBC. As revealed in Figure 2, the existence of peaks matches with the carbon (C), oxygen (O), iron (Fe), and silicon (Si) elements. The intensity of C peak is greater than O, Si, Al, and Fe, demonstrating the elevated content of C in NBC (Figure 2a). Also, three peaks displayed at 0.2, 0.5, and 6.40 keV to substantiate the binding energies of Fe_3_O_4_ nanoparticles [66]. The elemental composition of novel MBC was presented in Table 2 with the mass ratio of C, O, and Fe was 32.52, 27.23, and 38.14%, respectively.

The elements of NBC include C, O, Si, Al, and Fe, while the element observed in MBC were C, O, Si, and Fe. Noticeably, from Figure 2b,c, the Al element entirely disappeared after the magnetic modification, and also C, and Si weight components were significantly reduced. This indicates that chemical reactions occur during the modification. Conversely, Fe and O weight composition were substantially increased in modified MBC as compared to NBC. Particularly, the oxidation process considerably influences the obtainability of C active sites, in addition to the structural reformation of the adsorbent [67]. This indicates that the oxidation activity of the carbonaceous material justified the reduction in the weight composition of the trace elements, and also the formation of more acidic oxygen-containing functional groups on the surface [68]. These processes play a significant role in the development of more active sites for effectual adsorption process. The mass fraction of Fe in MBC was 38.14%. The high composition of Fe_3_O_4_ further proved the success of the magnetic modification of BC, which agreed with the FTIR and XRD analysis.

### 3.4. BET Surface Area Analysis of Biochar

**Figure 3:**
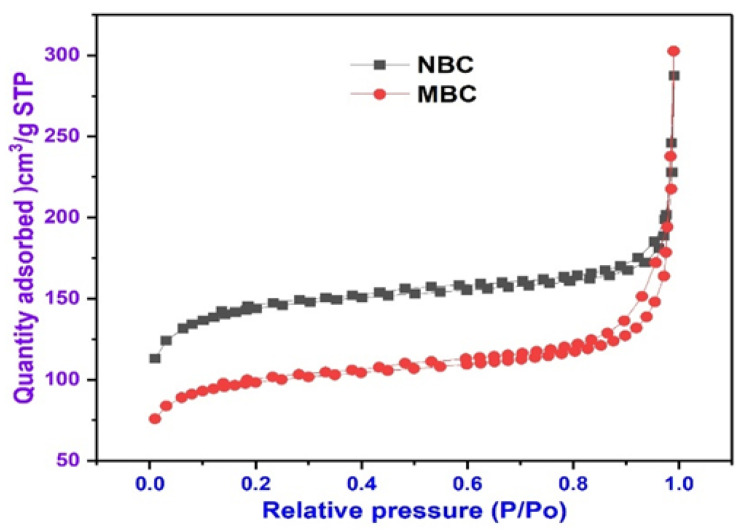
Nitrogen adsorption–desorption isotherm.

The nitrogen adsorption isotherm of NBC and MBC is presented in Figure 3. Essentially, the physical factors of BC samples that can influence the adsorptive removal of organic contaminants comprises of effective surface area, total pore diameter and pore volume. Surface area properties of the NBC and MBC were assessed via BET analysis. The BET results illustrate the adsorbate–adsorbent relationships of that adsorbed molecules which are gathered around on the surface of MBC [69]. The results of effective BET surface area, pore diameter and pores volume for both NBC and MBC are presented in Table 3. Noticeably from Table 3, the NBC exhibited a superior surface area of 536.54 m^2^/g, while MBC had a surface area of 362.07 m^2^/g. The decline in the surface area of MBC was possible since the MBC was comprised of a moderate surface area of Fe_3_O_4_ and an elevated surface area carbonaceous, and superfluous iron (Fe) nanoparticles loading could capture and clog some of the active sorption sites and pores of the NBC [70,71]. This reduction in surface area of MBC composites as compared to NBC has also been reported in previous studies [72,73]. Similarly, the pore size and pore volume of MBC are higher than that of NBC, which is better beneficial for the sorption of BPA.

Similarly, the total pore volume of NBC and MBC found were, respectively, 0.416260 cm^3^/g, and 0.442203 cm^3^/g, while MBC modified by Fe_3_O_4_ exhibits a better porosity, and presents a substantial capacity of pollutant adsorption, while after modification with Fe_3_O_4_, the existence of Fe_3_O_4_ between layers of MBC upsurges the heterogeneity of the adsorbent, thereby resulting in a superior porosity [74]. The impregnation of magnetite on BC has no significant influence on the pore-volume, surface area, and mean aperture. Iron-amendment can either decrease or increase the surface area of an adsorbent and this relies on the initial surface area value of the adsorbent and the proportions of the Fe_3_O_4_ particles [75]. The infrequent blocking of surface micropores from magnetite groups may also be responsible for the insignificant reduction in surface area. Thus, the adsorption capacity of modified biochar material is improved. This finding was further corroborated by the FESEM images and EDS spectra. Figure 3 displays the N_2_ adsorption–desorption isotherms of N_2_ at 77 K of NBC and MBC. The findings revealed that at moderately high relative pressures, the adsorption isotherm rose relatively than at relatively low pressures. This implies that the major adsorption takes place at moderately increased pressures and suggests that the material is highly porous with a narrow size distribution.

### 3.5. Magnetic Properties of As-Synthesized Magnetic Biochar

**Figure 4:**
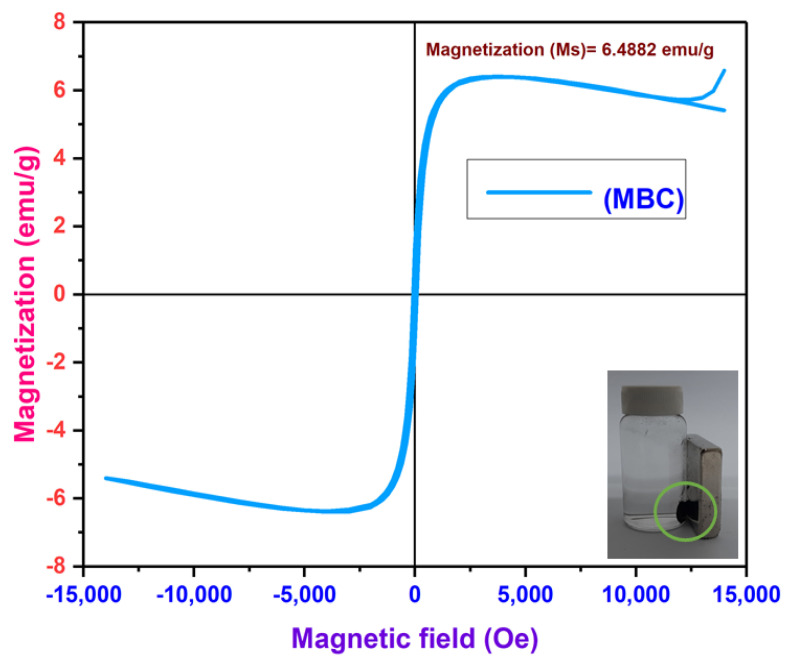
Magnetization curve for MBC.

The vibrating sample magnetometer (VSM) procedure was used to determine the hysteresis loop at room temperature. Most importantly it is employed to quantify the magnetic properties of a materials with respect to magnetic field, time, and temperature. Besides, VSM analysis provides information about whether the magnetization is perpendicular or parallel to the plane described by the substrate. The hysteresis loop of the synthesized MBC was obtained by plotting the magnetization (emu/g) against the magnetic field (Oe). Figure 4 reveals the magnetic hysteresis loops used to analyze the magnetic properties of MBC. As illustrated in the loop observed in Figure 4, the magnetization sharply increased with the decrease of the mass ration of BC. The curve signified distinctive super-paramagnetic properties. The synthesized MBC exhibited magnetization M values of 6.4882 emu/g which revealed that the magnetization of MBC can be altered by the mass ration of BC: magnetite (Fe_3_O_4_) nanoparticles. The super-paramagnetic properties was triggered by the alteration of BC with magnetite (Fe_3_O_4_) nano-particles, which could ensure the MBC can be readily recovered from the suspended solution through external magnetic field, which makes the replicated use of the MBC in the actual wastewater purification system viable [72]. Thus, novel MBC was easily detached using an external magnetic field, as illustrated by the inset. The result of magnetization obtained in this study is consistent with previous studies reported from [76,77,78].

### 3.6. XRD Analysis of Synthesized Biochar

**Figure 5:**
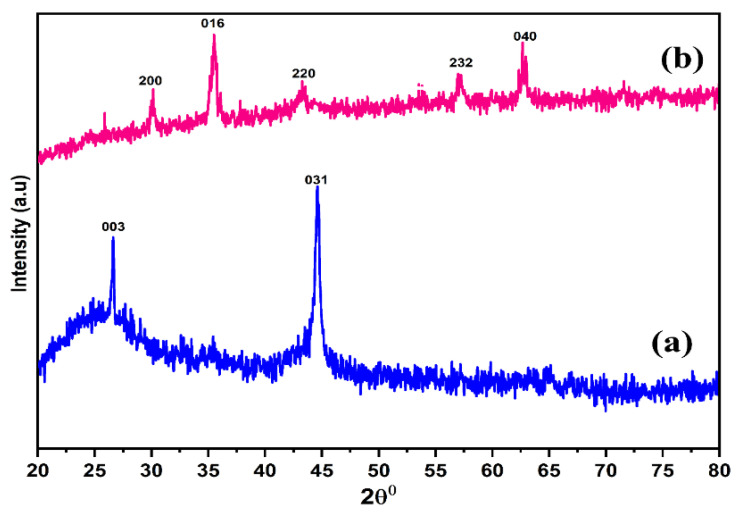
X-ray diffraction (XRD) patterns of NBC (**a**) and MBC (**b**).

X-ray diffraction analysis of a material describe the size and the nature of the planes of the synthesized material. The crystalline structures of NBC and MBC were analyzed using XRD as indicated in Figure 5. XRD is an efficient technique to verify the presence of Fe_3_O_4_ in the synthesized MBC [79]. Figure 5 reveals the XRD patterns of both NBC and MBC. The diffraction spectra of the synthesized BC samples showed the existence of magnetite (Fe_3_O_4_). The diffraction peaks at 2θ of 30.044°, 36.66°, 36.57°, 42.45°, 57.26°, 61.54°, and 62.76° are indexed to the (200), (016), (220), (232), and (040) hkL planes, respectively, which correspond satisfactorily with the database of Fe_3_O_4_ standard card Inorganic Crystal Structure Database (ICSD No. 98-007-7864) with a space group of P12/c1 and lattice parameter (a = b = c) of 28.644 Å and confirms the signature peaks of a hexagonal unit cell Fe_3_O_4_, respectively. No impurity peak is observed in the XRD pattern, which indicates that the Fe_3_O_4_ particles are highly crystalline hexagonal spinel structure. As observed from the spectra, all the diffraction peaks are designated to the magnetic hexagonal structure. There are no other peaks associated with another material detected from the XRD result, which confirmed that the nano-magnetite is pure magnetite (Fe_3_O_4_). The XRD spectra of Fe_3_O_4_ in this study is analogous to other studies from [80,81], and both Fe_3_O_4_ and BC patterns were overlapped in a XRD spectra of MBC, demonstrating successful synthesis of MBC composite.

### 3.7. Analysis of Functional Group

**Figure 6:**
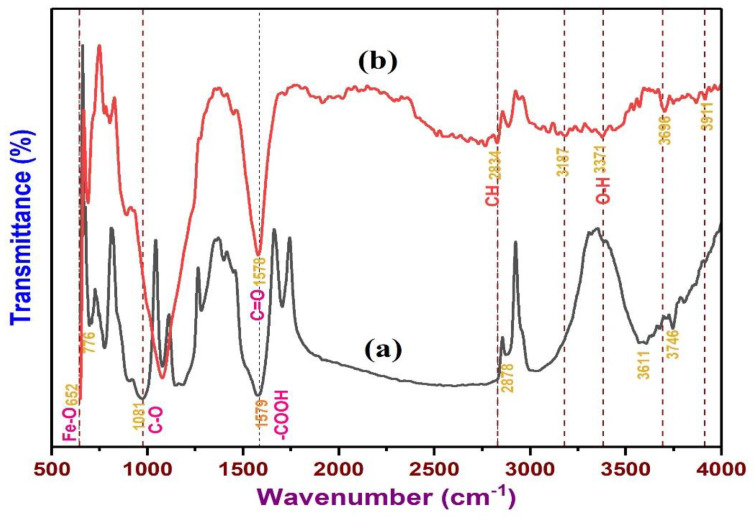
Fourier transform infrared spectra of NBC (**a**) and MBC (**b**).

The surface functional groups are the central chemical variable of BC material that influence the BPA adsorption. FTIR spectroscopy was employed to substantiate the modification process and acquire the information on the existence of different functional groups on the surface of the material [82]. The spectrum results and various bands in the spectra signifying vibration of functional groups were illustrated in Figure 6 for NBC and MBC, respectively. During the Fe^3+^/Fe^2+^/NaOH process, the surface of the char aids the nucleation of iron oxide precipitation. As indicated in Figure 6, the existence of functional groups of –COOH and –OH group are responsible for the binding of the iron oxide and iron hydroxide particles in the solution, and then bonded to the char [83]. This could be symbolized as char-O-Fe_x_O_y_ (Fe_3_O_4_/Fe_2_O_3_). In this context, the bold ‘O’ was initially a hydroxyl group on the surface and signify a chemical bond between iron oxide particle and the char phases. This bonding may conceivably be combined with some mechanical interlocking between H-bonding (such as between Fe–OH at the metal oxide surface with C–OH of the char surfaces) and phases in addition to some columbic interfaces. These interactions could strongly bind magnetic iron oxide to the BC materials [84].

Also, as indicated in Figure 6 of the FTIR spectra, there are limited peaks that surfaced for both NBC and MBC. The peaks at 2834, and 2878 cm^−1^ were distinctive peaks of the C-H bond [82]. Similarly, the peak at 1081 cm^−1^ was the C-O stretching vibration in the composition of carbohydrate, polysaccharide, or aromatic ether. The peaks close to 1578 and 1579 cm^−1^ were the stretching vibration peaks of C=C, C-H, and of C=O on the aromatic ring [85]. The results go along with the BC characteristics that not only contain porous structure but also abundant active adsorption sites for BPA removal [86]. Besides, a noticeable peak at 652 cm^−1^ was observed for MBC, which correspond to the typical stretching peak of Fe-O [87]. This indicated that the Fe_3_O_4_ nanoparticles have been efficaciously loaded on the surface of BC as revealed by the FESEM results. The spectra results revealed that magnetic alteration improved the varieties and number of functional groups of the BC, which may influence the adsorption of BPA.

### 3.8. Determination of Electrokinetic Charge (pH_pzc_) of Biochar

**Figure 7:**
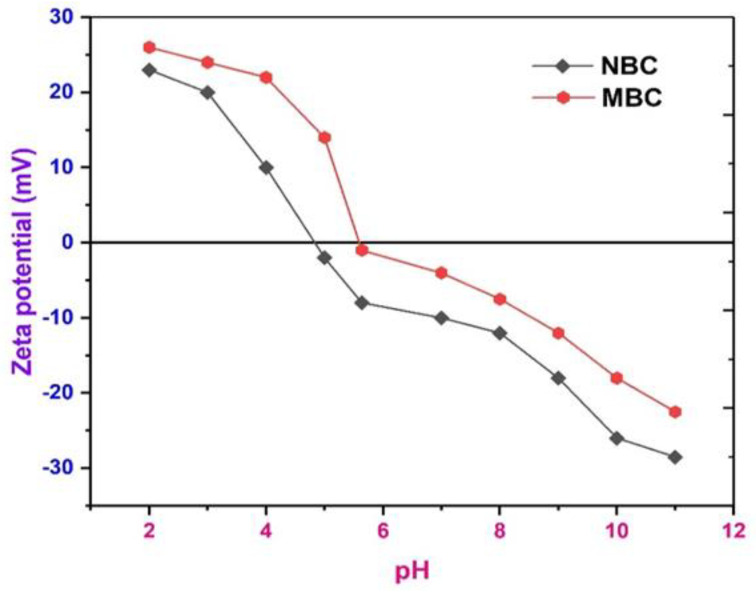
Zeta potential of NBC and MBC at different pH.

pH_pzc_ is the pH at which the net surface charge on the surface of an adsorbent is zero. It is a critical variable in examining the efficacy of the adsorption systems. When the pH is greater than the pH_pzc_, the adsorbent surface acquires negative charge, hence repelling or attracting organic contaminants, in line with their cationic or anionic functional groups [88]. The point of zero charges was investigated to describe the surface chemistry of MBC and NBC. The values of point of zero charge (pH_pzc_) of neat and MBC in relation to the solution pH were computed and displayed in Figure 7. According to Figure 7, the pH_pzc_ at the point where the change in pH (initial pH-final pH) equal to zero for the MBC and NBC were 5.61 and 4.81, respectively. It is observed that the pH_pzc_ of MBC (5.61) is higher than that of NBC (4.81). This increase may be due to the introduction of iron oxides (Fe_3_O_4_) on the surface of NBC [89]. Hence, higher pH_pzc_ is more beneficial for the adsorption of BPA anions, which are often existed in acidic solution [90]. The zeta potential of all samples steadily reduced with rising pH indicating more net negative surface charge at elevated pH. Similarly, the zeta potential of both NBC and MBC was both positive and negative within the whole pH range studied (2–11) as can be seen from Figure 7. Evidently, both MBC and NBC are positively charged when the pH is lower than 6.0. Once the pH is increase, the surface charge of the two biochar materials swiftly transformed from positive to negative. As pH consistently increase, the surface charges of both NBC and MBC risen and, noticeably, the zeta potential of NBC and MBC were −28.56 mV and −22.47 mV, which shows that both biochar samples were negatively charged (see Figure 7). Though, the surface of NBC is slightly more negatively charged, and the electrostatic attraction between the negatively charged NBC and HBPA improves the adsorption. This finding is in good agreement with previous studies [16,91].

### 3.9. Influence of Working Conditions on the Adsorption of BPA

The operating variables for instance contact time, solution pH, adsorbent dosage, ionic strength, BPA concentration, and other variables can influence the surface properties of the adsorbent surface and its BPA binding ability [88,92]. Hence, a batch adsorption test was carried out on the as-synthesized BC adsorbents to study the influence of these operating variables on the adsorption capacity and removal efficiency for BPA uptake.

### 3.10. Effect of pH on BPA Adsorption

**Figure 8:**
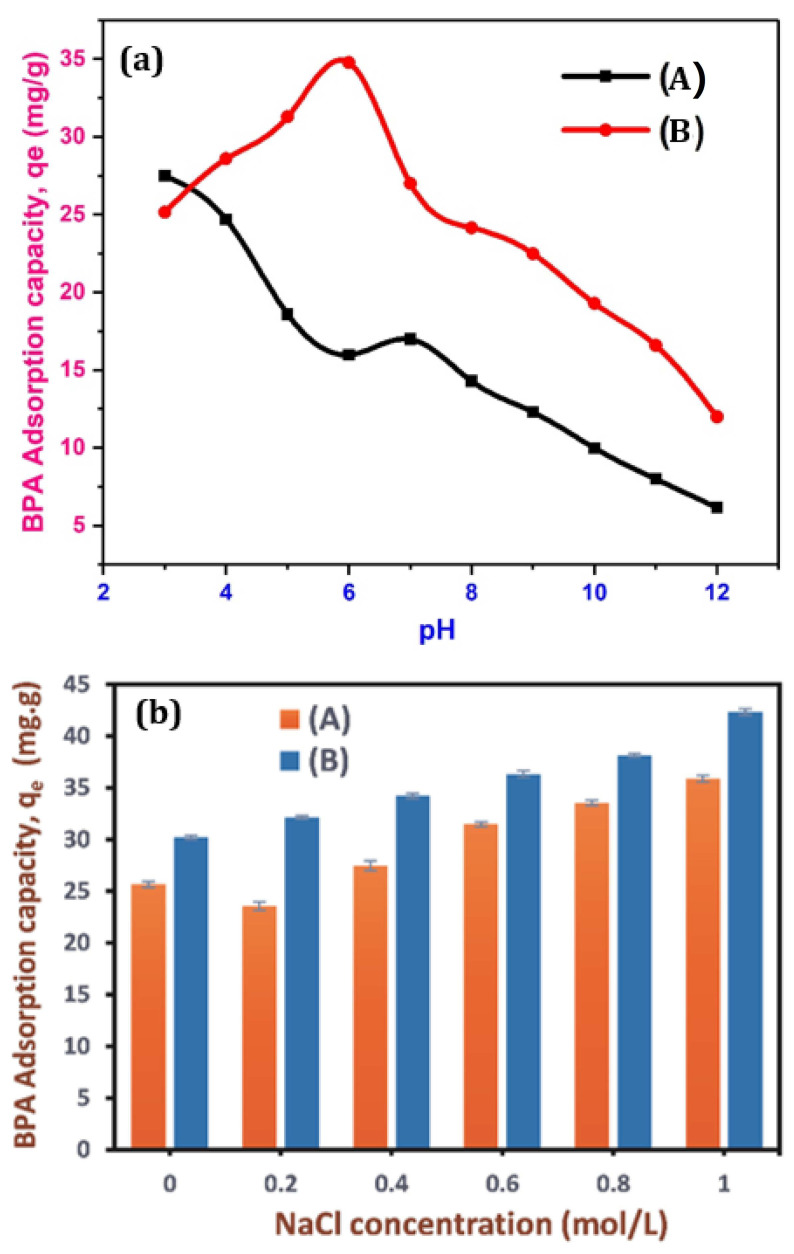
Effect of solution pH (**a**), and ionic strength (**b**) on the adsorption of BPA by NBC and MBC. (BPA concentration: 20 ppm; contact time: 60 min; biochar dose: 0.5 g; and temperature: 293.15 K).

The pH is one of the main parameters that control the removal of compounds present in aqueous environment using solid adsorbents. The solution pH is among the most essential variables that determine the elimination of various compounds existing in aqueous environment utilizing solid adsorbent materials and the optimization of adsorption process. The effect of pH on adsorption was reliant on the target contaminants and nature of absorbent [42]. It influences not only the speciation of the adsorbate, but also the level of ionization and adsorbent surface charge [90,93,94]. Accordingly, most of the investigations involved in contaminants adsorption onto BC strongly considered the effect of solution pH. To investigate the influence of different solution pH upon adsorption of BPA on the surface of NBC and MBC, the tests were performed in the pH range of 3.0–12.0, BPA concentration 20 ppm, adsorbent dose 0.5 g, and temperature 20 °C, and the results were illustrated in Figure 8a. As indicated in the figure, the sorption of BPA by NBC and MBC is clearly pH reliant. The highest adsorption capacity of NBC to BPA take place at pH (3.0), which is attributable to the development of electron receiver-giver interaction (ERG) between BPA and NBC, together with a robust hydrogen bond [95,96]. When pH rose from 6.0 to 7.0, the adsorption capacity of NBC to BPA slightly increased further, since BPA starts to moderately dissociate, and BPA in the solution is no more in molecular form, nevertheless few HBPA^−^ still occurs. The electrostatic attraction between NBC and HBPA^−^ enhances the adsorption process due to the positively charged surface of NBC. Further surge in pH led to a subsequent reduction in the adsorption capacity of NBC to BPA, owing to weakness in the interaction of the hydrogen bonding and π-π electron receiver–giver(ERG) between BPA and NBC when the solution pH is higher than the BPA acid dissociation constant [97]. Conversely, the adsorption capacity of BPA by MBC rose initially and after that it declined with the upsurge of pH greater than 6.0, which implied that the adsorption of BPA on BC was largely dependent on the solution pH of the system. Thus, the considerable rise in BPA adsorption on MBC between pH 3.0–6.0 was possibly attributed to the electrostatic interaction between the positively charged HBPA^+^ species and negatively charged surface of MBC in the solution. The surface of MBC may develop positively charged at low pH because of the protonation reaction (H^+^_(aq)_ + −ROH_(surf)_ ↔ −ROH^2+^_(surf)_) on the surface of MBC [98]. When the solution pH is increased, a negatively charged surface of MBC with plentiful active biding sites emerge owing to deprotonation reaction (OH^−^_(aq)_ + −ROH_(surf)_ ↔ H_2_O + ^–^RO^−^_(surf)_) on the surface of MBC. Hence, the enhanced BPA adsorption by MBC at pH 3.0–6.0 was not only controlled by the electrostatic interaction mechanism [99], but possibly caused by the surface reduction/complexation of BPA species onto MBC, and the decline of BPA adsorption at pH ˃ 6.0 following the repulsion interaction between negatively charged MBC and dissociated bisphenolate anions (BPA^2−^ and HBPA^−^) species. The maximum adsorption capacity take place at pH of 6.0.

### 3.11. Effect of Ionic Strength on BPA Adsorption

Generally, the water body comprises such a complex system that salts and organic contaminants frequently coexist in wastewater which may influence the removal of the contaminants. Hence, the profile of ionic strength influence on BPA adsorption by the as-synthesized BC was also investigated using 0–1.0 mol/L NaCl, BPA concentration 20 ppm, and the experimental findings are presented in Figure 8b. Initially, it could be noticed in Figure 8b, the increase in NaCl concentration led to a decline in the adsorption capacity of NBC to BPA and later a rise. Noticeably, the surge in high ionic strength (Na^+^ concentration) considerably increased the adsorption capacity of BPA to MBC (Figure 8b). The BPA adsorption capacity of BC increased when the Na^+^ concentration rose from 0 to 1.0 mol/L. Analogously, Zhou et al. [100], found that the upsurge in ionic strength with various ionic species (CaCl_2_ or NaCl) yielded increase in the adsorption capacity of BPA when peat was applied as an adsorbent. Similarly, erstwhile study has also reported that the increased ionic strength could improve the adsorption of organic contaminants into carbonaceous adsorbents, such as BC, owing to the screening effect of the surface charge generated by the addition of salt [101]. The BPA adsorption capacity by BC adsorbents rose steadily with the increase in the NaCl concentration. The plausible explanations for this surge could be due to the penetration of ions into the diffusion dual layer around NBC and MBC surfaces and lessen the repulsion between the adsorbents, thereby stimulating the squeezing-out effect (i.e., nanoparticle aggregation), which caused a decline in the adsorption capacity of BPA [102], enhanced activity coefficient of hydrophobic organic contaminants, which leads to a salting-out effect (reduction in solubility), and, therefore, was favorable to the adsorption of BPA [103]. Thus, the decreased adsorption capacity when the NaCl concentration is at 0.2 mol./L might be caused by the competition between BPA and the low concentration of salt solution for the accessible active sites of adsorbents, corroborating that the squeezing-out effect was stronger than the salting-out effect. Conversely, as the NaCl concentration increases, the adsorption capacity enhanced evidently, suggesting that the salting-out effect improved unceasingly [104].

### 3.12. Effects of Biochar Dose on the Removal of BPA

**Figure 9:**
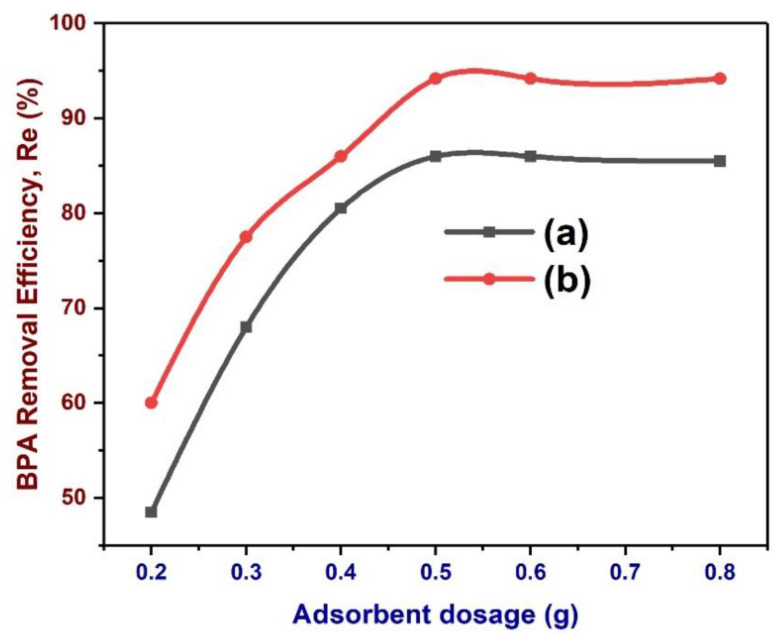
Effects of BC (a: NBC, b: MBC) dose on the removal of BPA. BPA removal efficiency by BC under varying doses: 0.2–0.8 g; BPA concentration: 20 ppm; temperature: 293.15 K; pH: 6.0; and contact time: 60 min.

Adsorbent dosage (AD) is an important parameter in an adsorption process. It controls the adsorbate–adsorbent equilibrium of the adsorption procedure. The removal efficiency based on the adsorbent dosage was studied at other test conditions, BPA concentration 20 ppm, varying dosages (0.2–0.8 g), pH 6.0, contact time (60 min), and adsorption temperature 20 °C. The influence of BC dose on the adsorption of BPA by NBC and MBC is illustrated in Figure 9. The removal rate of BPA enhanced with the rise in BC dose. When BC dosage was 0.2 g, the removal efficiencies of BPA by NBC and MBC were 48.45% and 60.0%, respectively. Similarly, when the BC dose rose to 0.5 g, the removal efficiency substantially rose to 85.97% and 94.2%, respectively. The increased removal rate is because of the augmented BC, which also expands the operational specific surface area of adsorption, together with increase in the active pore (binding) site of adsorption [105]. This outstanding adsorption performance was attributable to the presence of plentiful active sites and various interactions between BC adsorbent and BPA, specifically ion exchange and complexation electrostatic interaction. Afterward, further increase in BC dosage has no influence on the removal rate. This is because surplus adsorbent overlaps the effective active sites on BC and competes for limited solutes [42].

### 3.13. Effects of Initial Concentration and Contact Time on the Removal of BPA

**Figure 10:**
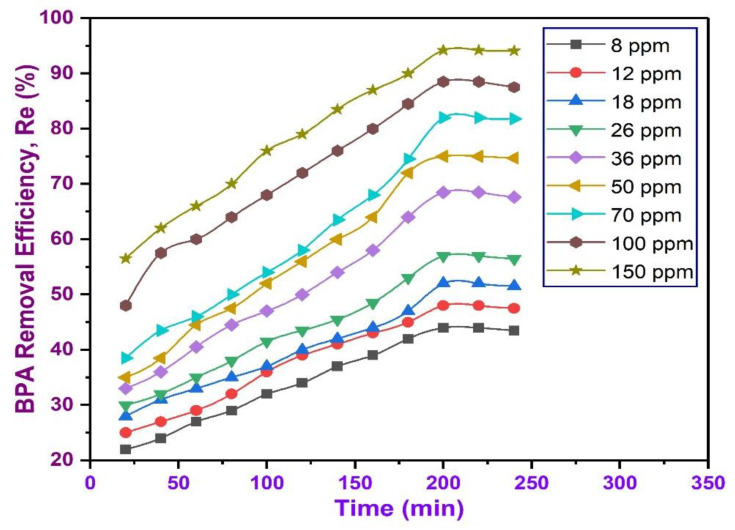
Effects of initial BPA concentrations on the removal efficiency under varying initial concentrations (8–150 ppm) at constant pH (6.0), dosage (0.5 g), and varying contact times (20–240 min).

The influence of initial BPA concentration and contact time on the adsorption of BPA from aqueous solution was investigated. The experiments were performed in the presence of a constant dose of adsorbent (0.5 g) of BC at room temperature, at pH (6.0) with various initial concentrations of BPA in the stock solution varied between 8.0 to 150.0 ppm, at different time intervals up to 240 min. The result of effect of initial concentration on BPA removal is presented in Figure 10. The initial concentration offers the stimulating force required to overcome the mass transfer wall between the adsorbate and the adsorbent media [106]. Thus, a higher initial concentration may enhance the efficacy of the adsorption process. Evidently, BPA removal is lower at a small concentration due to smaller amount of adsorbates in the solution to dominate active sites on the adsorbent and the amount of BPA adsorbed rises with the upsurge in BPA concentration. Thus, increase in initial BPA concentration, led to gradual rise in the removal efficiency of BPA, since the increased BPA concentration can improve the adsorption drive between the solute and the adsorbent [107,108]. The rise in the concentration of BPA conforms with an increase in BPA removal as shown in Figure 9. When equilibrium is attained, the adsorbent turns out to be saturated. This result is in line with previous study reported by Wang and Zhang [16].

**Figure 11:**
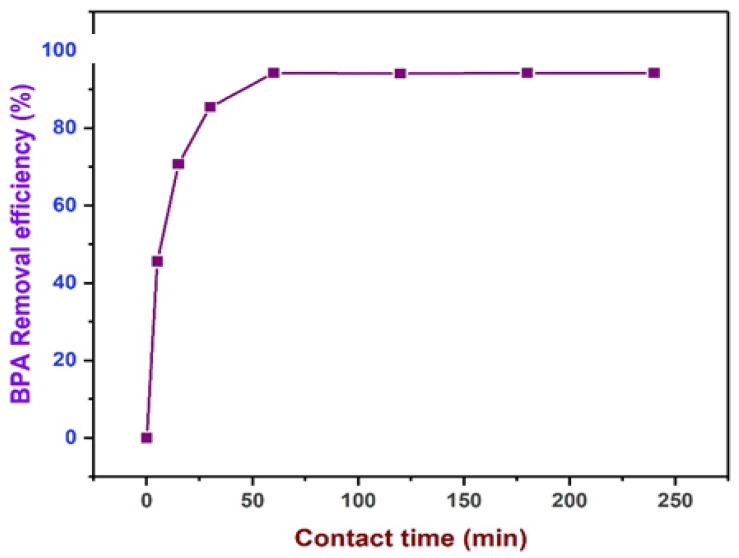
Effects of contact time (20–240 min) on the per cent removal of BPA, at 0.5 g dosage, BPA concentration 20 ppm, temperature (293.15 K), and pH (6.0).

The test conditions evaluating the influence of contact time on the adsorption of BPA at the test conditions, BPA concentration 20 ppm, adsorbent dosage 0.5, and pH 6.0. Similarly, the result of the effects of contact time on the adsorption capacity and the removal efficiency are illustrated in Figure 11. Noticeably, more than 93% of BPA became adsorbed in about 60 min. For contact time beyond 60 min, the per cent removal of BPA remains stable, because the active sorption sites have been saturated on the adsorbent surface.

### 3.14. BPA Sorption Isotherm Study

Sorption isotherm models are indispensable for recognizing the mechanisms of the adsorption process based on fundamental characteristics and numerical derivations. Also, the isothermal models are used to study the interrelatedness between the adsorbate and the adsorbent. The Freundlich and Langmuir isotherm models are normally employed and were fitted to the adsorption experimental data. The Freundlich isotherm is appropriate to both multilayer and monolayer adsorption and assumes that the adsorbates are adsorbed onto the heterogeneous surface of an adsorbent [109]. The Langmuir isotherm assumes monolayer adsorption on a uniform surface with a limited amount of adsorption sites [110].

**Figure 12:**
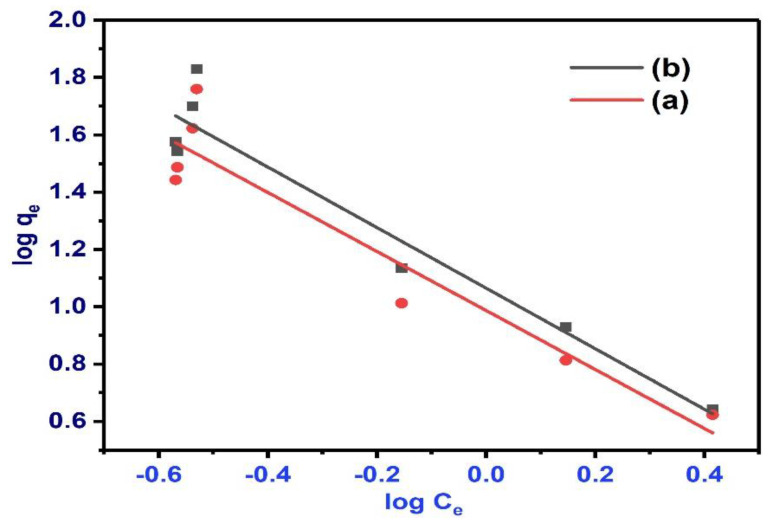
Freundlich isotherm for BPA adsorption onto NBC (a) and MBC (b) at pH 6.0, dose 0.5 g, contact time 240 min, and initial BPA concentration 50 ppm.

**Figure 13:**
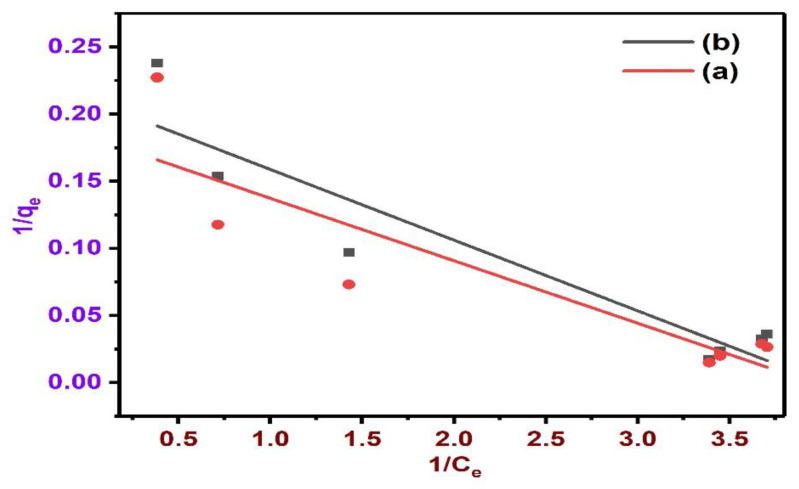
Langmuir isotherm on BPA adsorption onto NBC (a) and MBC (b) at 0.5 g dose, pH 6.0, contact time 240 min, and initial BPA concentration 50 ppm.

The Langmuir isotherm model presumes that every molecule has a stable adsorption enthalpy and activation energy and signifies homogeneous adsorption. Correspondingly, the Freundlich isotherm model is experimental and regards the surface to be heterogeneous [111]. Two regular isotherm models were applied to fit the BPA adsorption isotherms on the as-synthesised BC (Figure 12 and Figure 13). The models were employed to better investigate the BPA adsorption mechanism and performance. The fitting data are illustrated in Figure 12 and Figure 13, and the applicable fitting parameters of these models were summarized in Table 3. The BPA adsorption experimental data were fitted to the Freundlich and Langmuir isotherm models with Origin 8.0.

The fitting of the experimental data into the isotherm models illustrates the adsorption process by the correlation coefficient R^2^ and constants. To accomplish this, log *q_e_* is plotted against log C_e_ based on the linear form of the Freundlich model (Figure 12) [112]. For the Langmuir model linear expression, 1/*q_e_* versus 1/*C_e_* is plotted (Figure 13) [113,114]. The computed q_max_ values of the Langmuir model from the graph of 1/*q_e_* against 1/*C_e_* was lower than the *q_e_* values from the experiments (see Table 4), indicating that the experimental adsorption data fit this model. However, the R^2^ value of Langmuir is lower than that of the Freundlich model.

The variables revealed that the Freundlich model (R^2^ = 0.88964–0.9195) offers a superior fit to the data than the Langmuir model (R^2^ = 0.7535–0.85608), as showed by the linear regression values. Additionally, the heterogeneity coefficient (1/n) of the two BC falls above 1 (1/n ˃ 1), which implied satisfactory physical adsorption process [115]. This trend was also corroborated by several studies [116,117] using BC synthesized from eucalyptus forest residues, sugarcane bagasse, castor meal, water hyacinth, green pericarp of coconut, and MBC to remove various contaminants. Hence, BC generated from various biomasses at different temperatures were observed to have various adsorption mechanisms and adsorption abilities [118]. In this work, BPA adsorption onto NBC and MBC was excellently fit using the Freundlich model in the determination of the efficacy of NBC and MBC for the removal of BPA, which demonstrates that the adsorption take place as a heterogeneous surface multilayer. A synopsis of the isotherm variables is presented in Table 4.

### 3.15. BPA Adsorption Kinetic Studies

Kinetic study offers essential information on the mechanism of adsorption and influencing mechanism of adsorption process as either chemical reaction or mass transfer to attain optimal working conditions for industrial scale [119]. With a view to examine the kinetic mechanism triggering BPA adsorption, experimental data were fitted to the pseudo-first order (PFO) [120] and pseudo-second order (PSO) [121] linearized models using Equations (4) and (5), respectively:

(4) $$ln\left(q_{e}-q_{t}\right)=ln\left(lnq_{e}-K_{1}\;t\right)$$

(5) $$\frac{1}{q_{t}}=\frac{1}{K_{2}q_{e}{}^{2}}\times \frac{1}{t}+\frac{1}{q_{e}}$$

where *q_t_* and *q_e_* are the amounts of BPA adsorbed at time t and equilibrium, respectively (mg/g), t is the contact time (min), and *K*_1_ and *K*_2_ are the rate constants of the PFO and PSO kinetic models, respectively (1/min). In batch process, sorption kinetics is described by different models based on adsorption equilibrium including the PSO (pseudo-first order) and PSO (pseudo-second order) kinetic models. In this context, it is used to model the kinetics of the adsorption of BPA onto BC.

**Figure 14:**
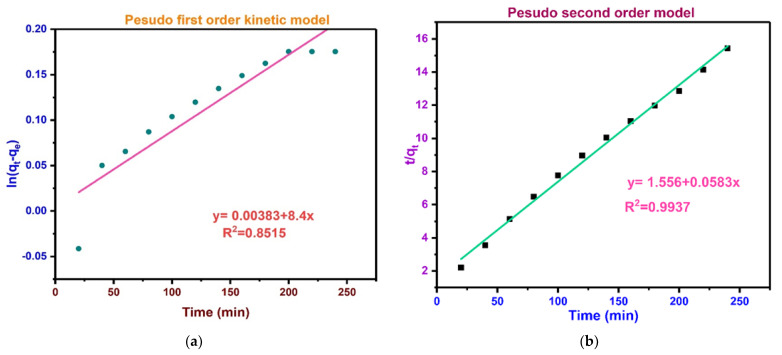
(**a**) Pseudo-first order model for adsorption of BPA by MBC, and (**b**) pseudo-second order model for adsorption of BPA by MBC.

Figure 14a,b illustrates the graphs of (*ln*(*q_e_* − *q_t_*) against time) and (time/*q_t_* against time) for the pseudo-first order and pseudo-second order kinetic models, respectively. The computed variables and the experimental data of the two models are presented in Table 5. Noticeably the coefficient of correlation value (R^2^ = 0.9937) for the PSO model is of better linearity and higher in comparison with the PFO (R^2^ =0.8515) for BPA adsorption, which was also noticed for a similar compound (sulfamethoxazole) adsorption by functionalized BC [122]. Similarly, the computed *q_e_* (mg/g) for the PSO model is close to the experimental *q_e_* value (see Table 5). Hence, PSO model satisfactorily fits better the experimental results than the PFO kinetic model. The PSO kinetic model suggests that the chemisorption can be regarded as a rate-controlling phase during the adsorption procedure [3,123]. Similarly, the chemisorption takes place via electron-exchange or sharing between BPA and BC [124]. The elevated rate constants of the PSO model for BPA can be attributed to rapid interaction with the active adsorption sites of MBC. This finding agrees with previous studies on the BPA adsorption on the following adsorbents: algal BC [48], magnetic CuZnFe_2_O_4_–BC composite [125], modified organo-montmorillonites [126].

### 3.16. Adsorption Thermodynamics

Thermodynamic studies are employed to decipher any reaction in a better approach and reveals whether the adsorption process is an intended process or a spontaneous process [127]. It also indicates the influence of temperature on the adsorption process. Generally, exothermal and endothermal sorption processes are the two common processes. If the sorption declines with increasing temperature, it implies the exothermal sorption process whereas if the sorption increases with rising temperature, it signifies that the sorption is an endothermal process. In this study, the temperature influence on BPA adsorption onto MBC was studied by performing adsorption tests at varying temperatures, viz., 298.15, 318.15, and 338.15 K, at optimum pH (6.0) and adsorbent dosage 0.5 g, respectively. The thermodynamic variables such as enthalpy change (Δ*H*°), standard Gibb’s free energy change (Δ*G*°), and change in entropy (Δ*S*°), were computed and summarized in Table 6 using the following equations:

(6) $$\Delta G_{O}=-RT\ln K_{L}$$

**Figure 15:**
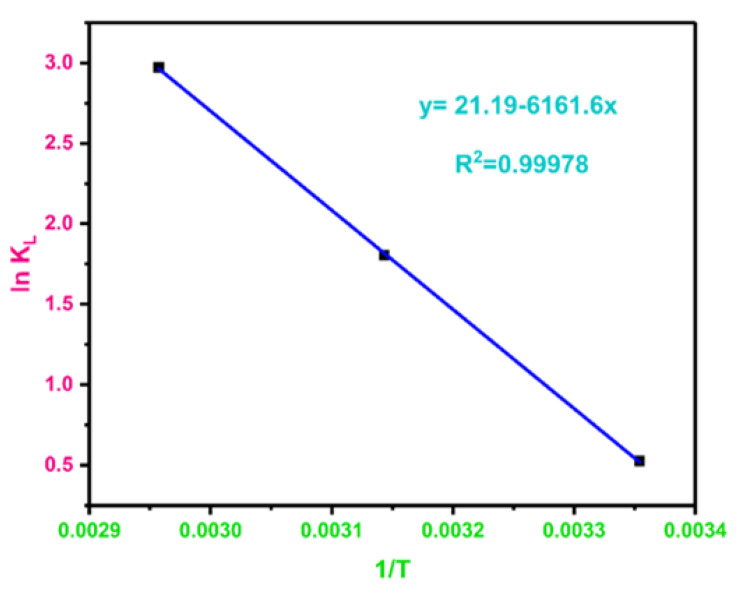
Thermodynamic plot of lnK_L_ versus 1/T for adsorption of BPA on MBC.

(7) KL=qeCe

(8) $$\Delta S^{{}^{\circ}}=\Delta H^{{}^{\circ}}-\Delta G^{{}^{\circ}}/T$$

where *R* is the gas constant (8.314 J/mol^−1^K^−1^), *q_e_* is the adsorption capacity (mg/g), *C_e_* is the equilibrium concentration (mg/L) and T is the actual temperature (*K*). The y-intercept and the slope of the linear fit following a plot of ln *K_L_* versus 1/T were employed to calculate the values of Δ*H°* and Δ*S°* via the Van’t Hoff plot (Figure 15).

As the sorption temperature rises, the values of Δ*G°* turn out to be more negative, which implies that BPA is better effectively adsorbed to MBC at elevated temperatures. The negative Δ*G°* indicate that all adsorption processes were spontaneous [128]. The positive Δ*H°* (51.23 kJ·mol^−1^) indicated that the interaction between BPA and MBC was an endothermic process which possibly attributed to the competitive dissolution of BPA in aqueous solution [129]. The positive Δ*S°* and negative ΔG0 values suggested the spontaneity of the adsorption process. The higher positive Δ*S°* (176.13 J·mol^−1^·K^−1^) as indicated in Table 5, further demonstrate increasing randomness at the adsorbate-adsorbent interface as a result of free water molecules [130]. Thus, the determined positive Δ*S°* and negative Δ*G°* values at experimented temperatures substantiated the BPA adsorption on MBC and indicated the spontaneity of the sorption process, besides the entropy effect should be the primary leading force for the adsorption of BPA on MBC [131].

### 3.17. Investigation of Biochar Regeneration and Reusability

**Figure 16:**
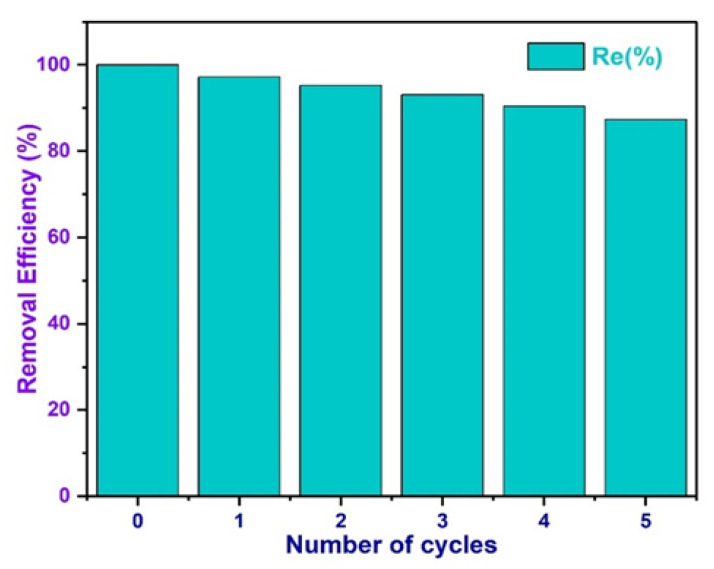
Regeneration of magnetic biochar for BPA removal. Experimental conditions: m = 0.5 g; [BPA] = 20 ppm; and temperature (293.15 K).

Recyclability of the adsorbents is strongly essential to preserve process costs down and for the industrial-scale application. Five phases of adsorption–desorption tests were undertaken to examine the reusability of the as-synthesized MBC in accordance with previously adopted experimental procedure [125]. The MBC was repetitively applied five times, and the *q_e_* values were recorded, as illustrated in Figure 16. The result indicated that *q_e_* was slightly reduced during the adsorption–desorption experiment. Upon the completion of five cycles of reusability test, the removal efficiency of BPA only reduced by 12.85% as compared with the first cycle. The slight reduction in adsorption capacity during multiple adsorption–desorption tests, was due to the partial desorption of BPA in MBC surface, competitive available sorption sites, loss of solid in solution, and the elution of iron oxide nanoparticles and energetic substances on MBC surface [49,132]. This reveals that the MBC could be an effectual, economical, and environmental benign adsorbent with superior re-usability, which can be practically applied in BPA removal procedures. The recyclability test reveals that MBC can be applied repeatedly in wastewater purification as an efficient adsorbent.

Table 7 presents the comparison of specific surface area characteristics, adsorption capacity of BPA, and the magnetic intensity of the synthesized MBC material in the current study in comparison with various studies.

Though few studies reported higher adsorption capacity than the current study despite their low surface area as indicated in Table 7, this is because previous studies have described the performance of MBC to be significantly influenced by the nature of auxiliary and raw materials, starting contaminant concentration, pyrolysis temperature, competitive anions, nature of modifier, reaction temperature, sorption time, and various synthesis methods [86,136,137]. As highlighted in Table 7, the maximum adsorption capacity recorded in the current study is comparable and in line with previous studies using different BC adsorbents. Hence, it can be inferred that the MBC synthesized from palm kernel shell is a promising, efficient, and essential precursor (adsorbent) for the removal of BPA from aqueous environment. The sorption capacity recorded from the current study using magnetic PKS-BC could be stems from highly developed pore structure, superfluous surface area, smaller pore size, considerable surface functional group, high sorptive capacity, and super paramagnetism. It is noteworthy that studies on the adsorption of BPA onto MBC synthesized in-house from palm kernel shell biomass is still very limited. Besides, the adsorbent demonstrated superior regeneration efficacy, and the resultant MBC could be reclaimed numerous times.

### 3.18. Controlling Mechanism for BPA Removal

**Figure 17:**
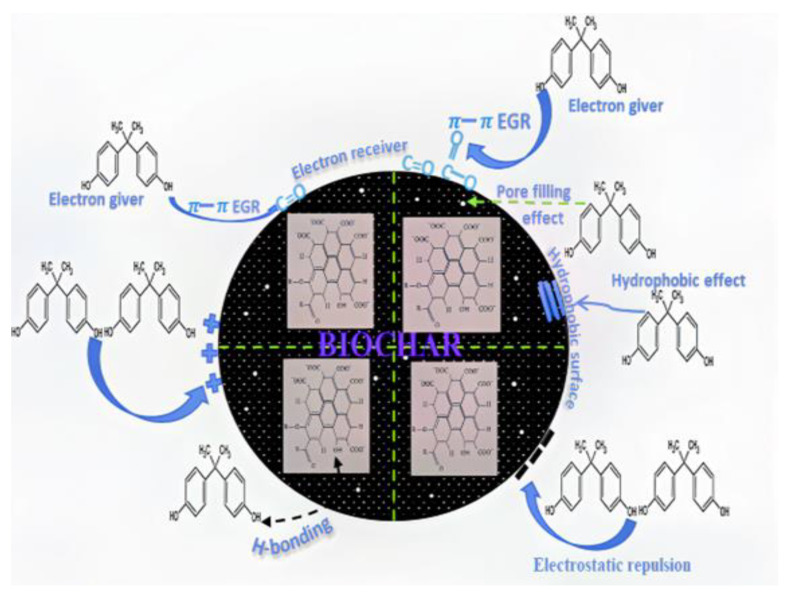
Schematic coordination of BPA removal mechanism using biochar.

The schematic diagram of mechanisms of adsorption of BPA (adsorbate) onto as-synthesized NBC and MBC is depicted in Figure 17. The average pore size of the as-synthesized BC falls below 50 nm, indicating mesoporous. Also, BPA molecules can penetrate the pores of the as-synthesized BC via pore filling. As stated earlier, the adsorption capacity of NBC and MBC to BPA is largely influenced by pH. In acidic environments, the phenolic hydroxyl groups in the chemical structure of BPA protonate, and thereby producing electrostatic repulsion with the positively charged surfaces of both NBC and MBC. Also, since BC adsorbent comprises carbon, silicon, iron, and oxygen groups, these elements C, Si, Fe, and O groups are vastly electronegative because of the sufficiency of available lone pair of electrons, which exhibits binding capabilities toward the BPA as indicated in Figure 2. The carboxyl, hydroxyl, carbonyl, and amine functional groups (as identified in Figure 5) facilitate the affinity of BPA molecules and their adsorption on the surface of BC. Such sorption mechanism is an electron giver–receiver type according to the unbalanced electrons’ supply between BPA compound and the BC functional groups. The hydroxyl functional group (^−^OH) on the BC’s surface also develops potent hydrogen bonds with the C-H and ^−^OH on the molecular composition of BPA. Also, the C=O and ^−^COOH acidic functional groups of the as-synthesized BC can serves as electron receivers, producing π-π electron giver–receiver interactions (EGR) with BPA. In alkaline environments, the surface of MBC becomes negatively charged, which retains the electrostatic repulsion with the dissociated BPA^2−^ and HBPA^−^, whereas NBC acts oppositely. Similarly, the π-π electron giver-receiver interaction (EGR), as well as hydrogen bonds between BPA and as-synthesized BC, would be severely weakened, thereby making it hard for BC to adsorb BPA. Since, BPA exhibits robust hydrophobicity and can be intermixed with the hydrophobic site on NBC and MBC surfaces. Hence, a hydrophobic interaction is correspondingly a central influential force for the adsorption of BPA onto NBC and MBC.

## 4. Conclusions

In this work, a novel magnetic palm kernel shell biochar was efficiently synthesized via magnetic modification and applied to remove BPA from aqueous solution. The results of VSM, XRD, FTIR, BET, FESEM/EDX, and Zeta potential revealed that the Fe_3_O_4_ nanoparticles was effectually engrafted on the surface of biochar. The experimental findings demonstrated the adsorption isotherm could be better fitted by Freundlich model, whereas the adsorption kinetic data was controlled by pseudo-second order model. The as-synthesized BC also demonstrated a good magnetic strength for facile recovery, superior recyclability, high selectivity, and eco-friendly. It was noticed that BPA removal is greatly influenced by adsorbent dose, pH, and contact time. The BPA adsorption does increases with reduction of pH, with a maximum adsorption at pH 6 for MBC. Thermodynamic study revealed that BPA adsorption on MBC is endothermic process and spontaneous due to +ΔH and −ΔG, respectively. The principal mechanisms for BPA adsorption on the prepared BC comprised of electrostatic interactions (π-π electron acceptor– donor interactions), hydrophobic interaction, and H-bonding. Therefore, it can be deduced from this study that novel MBC is efficient and practical for the removal of BPA from aqueous solution with benefit of being sustainable, and abundantly available.

## Figures and Tables

**Table 1:** Properties of Bisphenol A (BPA).

Compound Name	Lipid-Water Partition Coefficient (Log K_ow_)	Molecular Mass (g/mol.)	Chemical Structure	Molecular Formula	pKa
Bisphenol A	3.32	228	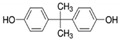	C_15_H_16_O_2_	10.1

**Table 2:** Elemental constituents of magnetic biochar.

Elements	C	O	Si	Fe	Total
Mass ratio (%)	32.52	27.23	2.11	38.14	100.00
Atom ratio (%)	52.40	32.93	1.45	13.22	100.00

**Table 3:** Physico-chemical properties of neat biochar and magnetic biochar.

Materials	Pore Diameter (Å)	Pore Volume (cm^3^/g)	BET Specific Surface Area (m^2^/g)	pH_pzc_
NBC	15.516	0.416260	536.5398	4.829
MBC	24.427	0.442203	362.0673	5.612

**Table 4:** Adsorption Isotherms variables.

Adsorbents	Freundlich Model		Langmuir Model				
	1/n	*K_F_*(L·mg^−1^)	R^2^*	q_max_(mg/g)	*K_L_*(L·mg^−1^)	R_L_	R^2^**
NBC	1.02974	9.718964	0.88964	5.438033607	3.95207	0.00509	0.7535
MBC	1.05679	11.62947	0.9195	4.72701489	4.01652	0.005	0.85608

Note: 1/n, *K_F_*, and R^2^* were computed from a plot of log*q_e_* versus logC_e_ (Equation (5)), while *K_L_*, qmax, and R^2^** were calculated by plotting 1/*q_e_* against 1/C_e_ (Equation (4)), respectively, using the data from Figure 10 and Figure 11.

**Table 5:** Kinetic study result for BPA adsorption.

Kinetic Models	Parameters	Value	Linear Regression
PSO $\frac{1}{q_{t}}=\frac{1}{K_{2}q_{e}{}^{2}}\times \frac{1}{t}+\frac{1}{q_{e}}$ where *K*_2_ is rate constant (g·mg^−1^·min^−1^)	*K*_2_ (g·mg^−1^·min^−1^)	0.005289	y=1.556+0.0583x
	Comp. *q_e_* (mg/g)	47.15266	
	Exp. *q_e_* (mg/g)	37.75	
	R^2^	0.99376	
PFO ln(*q_e_* − *qt*) = ln(*q_e_*) − *K*_1_*t*	*K*_1_ (min^−1^)	3.50 × 10^−6^	y=0.00383+8.4x
	Comp. *q_e_* (mg/g)	12.003837	
	Exp. *q_e_* (mg/g)	37.75	
	R^2^	0.8515	

NB: PSO is pseudo-second order; PFO is pseudo-first order.

**Table 6:** Computed thermodynamic variables for BPA adsorption on MBC at varying temperature.

Adsorbate	Temperature (K)	lnK_L_	Δ*G°* (kJ·mol^−1^)	Δ*H°* (kJ·mol^−1^)	Δ*S°* (J·mol·K^−1^)
BPA	298.15	0.526093278	−1.304090065	51.22768066	176.1349999
	318.15	1.803593997	−4.770684859		
	338.15	2.971634746	−8.354391819		

**Table 7:** Comparison of specific surface area, magnetic strength, and adsorption capacity of BPA on different adsorbents from previous literature with current study.

Adsorbents	Surface Area (m^2^/g)	Magnetic Strength (emu/g)	Regeneration (%)	Adsorption Capacity (mg/g)	Reference
ulva prolifera (marine macroalgae)	25.43	ND	ND	84.19	[48]
pomelo peel	889.8	ND	ND	26.25	[133]
magnetic composite sepiolite	NA	14.1	NA	36.30	[134]
bamboo	61.5	37.6 and 32.6	7.6 and 8.2	263.2	[125]
sewage sludge					
wheat straw	65.03	ND		196.91	[135]
dried pineapple	84.89	12.83	34.93	101.16	[47]
corn straw	313.88	14.5	ND	46.90	[111]
local reed biomass	154.79	ND	ND	9.92	[136]
grapefruit peel	20.732	30.60	20	229.19	[16]
magnetic biochar palm kernel shell	362.0673	6.4882	12.85	37.64	Current study

NA = Not Available; ND = Not determined.

## Data Availability

The data presented in this study are available on request from the corresponding author.
